# The Subcellular Localization and Oligomerization Preferences of NME1/NME2 upon Radiation-Induced DNA Damage

**DOI:** 10.3390/ijms21072363

**Published:** 2020-03-29

**Authors:** Martina Radić, Marko Šoštar, Igor Weber, Helena Ćetković, Neda Slade, Maja Herak Bosnar

**Affiliations:** 1 Division of Molecular Medicine, Ruđer Bošković Institute, Bijenička 54, 10000 Zagreb, Croatia; Martina.Radic@irb.hr (M.R.); slade@irb.hr (N.S.); 2 Division of Molecular Biology, Ruđer Bošković Institute, Bijenička 54, 10000 Zagreb, Croatia; Marko.Sostar@irb.hr (M.Š.); iweber@irb.hr (I.W.); cetkovic@irb.hr (H.Ć.)

**Keywords:** NME, NDPK, Nm23, nucleoside diphosphate kinase, FRET, FLIM, live-cell imaging

## Abstract

Nucleoside diphosphate kinases (NDPK/NME/Nm23) are enzymes composed of subunits NME1/NDPK A and NME2/NDPK B, responsible for the maintenance of the cellular (d)NTP pool and involved in other cellular processes, such as metastasis suppression and DNA damage repair. Although eukaryotic NDPKs are active only as hexamers, it is unclear whether other NME functions require the hexameric form, and how the isoenzyme composition varies in different cellular compartments. To examine the effect of DNA damage on intracellular localization of NME1 and NME2 and the composition of NME oligomers in the nucleus and the cytoplasm, we used live-cell imaging and the FRET/FLIM technique. We showed that exogenous NME1 and NME2 proteins co-localize in the cytoplasm of non-irradiated cells, and move simultaneously to the nucleus after gamma irradiation. The FRET/FLIM experiments imply that, after DNA damage, there is a slight shift in the homomer/heteromer balance between the nucleus and the cytoplasm. Collectively, our results indicate that, after irradiation, NME1 and NME2 engage in mutual functions in the nucleus, possibly performing specific functions in their homomeric states. Finally, we demonstrated that fluorophores fused to the N-termini of NME polypeptides produce the largest FRET effect and thus recommend this orientation for use in similar studies.

## 1. Introduction

Nucleoside diphosphate kinases (NDPKs) are ubiquitous enzymes which catalyze the transfer of the terminal phosphate group from (d)NTPs to (d)NDPs through a high-energy phosphohistidine intermedier [1]. These enzymes are considered to be key players in the maintenance of the cellular NTP pool. The NDPKs are encoded by *NME/nm23* genes. Ten NME genes/proteins have been identified in humans so far, but only NME1-NME4 proteins have a confirmed NDPK activity [2]. It is generally accepted that eukaryotic NDPKs are active only in the form of hexamers, while the prokaryote enzymes display a tetrameric structure [3]. In humans, at least 80% of the cytoplasmic NDPK activity is being exerted by NME1/NDPK A and NME2/NDPK B, which combine to form a series of homo- or heterohexameric isoenzymes (A6, A5B1, B6) [4]. NME1 and NME2 are highly homologous in their nucleotide and amino acid sequence and possess the same gene and protein architecture. The two genes are located in tandem on chromosome 17q21.3 (http://www.ncbi.nlm.nih.gov/Genbank) and are highly homologous to their orthologs in rodents [5]. *NME1* and *NME2* separated through cis-duplication from a common ancestral gene after the emergence of amphibians and are considered to be paralogs [6]. Both NME1 and NME2 possess the NDP kinase active site motif (NXXHG/ASD) and are enzymatically active with similar kinetic parameters [7]. However, their polypeptide chains differ in 18 out of 152 amino acid residues, making NME1 an acidic and NME2 a basic protein. A huge interest for these enzymes was raised in the early 1990s when it was suggested that the gene encoding NME1 was responsible for metastasis suppression in a murine melanoma model system [8]. In the years to come, a number of studies reported that downregulation or loss of expression of *NME1* was correlated with the onset of metastasis and poor clinical outcome in many tumor types such as melanoma, breast cancer, ovarian carcinoma, hepatocellular and laryngeal carcinoma, and several other malignancies [9]. Although not studied so extensively, NME2 was also shown to exhibit metastasis suppressor activity to some extent [10]. However, in spite of intense research in this area, we still do not have a mechanistic model of metastasis suppression activity of NME proteins.

Numerous studies reported that NME1 and NME2 participate in various major cellular processes such as proliferation [11,12,13], apoptosis [14,15], differentiation and development [16,17,18,19], adhesion and migration [20,21], and vesicular trafficking [22,23,24]. While the NDPK activity of NME1/NME2 is well documented and mechanistically straightforward, both proteins have been appointed additional biochemical activities, together or individually, which arose numerous still unanswered questions. Histidine protein kinase activity has been assigned to both enzymes, but it was shown to target different proteins. NME1 phosphorylates aldolase C [25], ATP citrate lyase [26], and kinase suppressor of Ras, KSR (Kinase Suppressor of Ras), and this was hypothesized to be one of the major mechanisms of NME1-mediated metastasis suppression [27]. NME2 phosphorylates the Gβ subunit of heterotrimeric G-proteins on histidine 226 [28], and K_Ca_3.1 potassium channel on histidine 358 [29]. A transcriptional regulatory function has also been reported for both proteins: They both appear to repress the transcriptional activity of PDGF promoter, while NME2 additionally plays the role of a transcription factor for c-*MYC* oncogene [30], a finding that has been challenged by other authors [31]. Other DNA-based activities of NME proteins include the 3′-5′exonuclease activity of NME1 that potentially contributes to the regulation of metastatic potential [32], and the endonuclease activity of NME1 within a macromolecular complex associated with the endoplasmic reticulum and targeted by Granzyme-A during cytotoxic T lymphocyte-induced apoptosis [33]. Numerous studies reported NME1 and NME2 to be associated with a number of different binding/interacting partners [34]. Both proteins have been found to interact with dynamin [24], while NME1 additionally interacts with PRUNE 1 (Prune Exopolyphosphatase 1) [35], STRAP (Serine/Threonine Kinase Receptor Associated Protein) [36], MIF (Macrophage Migration Inhibitory Factor) [37], VHL (Von Hippel–Lindau) tumor suppressor [38], tumor virus-encoded oncoprotein EBNA 1-3C (Epstein-Barr Nuclear Antigen 1–3C) [39], and a number of small GTPases [40]. NME2 was reported to interact with MDM2 (Mouse Double Minute 2 Homolog) [41], TRF1 (Telomeric Repeat Binding Factor 1) [42], ICAP1α (Integrin Cytoplasmic Domain-Associated Protein 1α) [21], and several other proteins. *NME1* and *NME2* are ubiquitously expressed, but the level of expression varies among different tissues and cell lines. Since it has been reported that their promoter regions contain binding sites for distinct transcriptional factors, it is presumed that the two paralogs might be regulated dissimilarly and in a cell-specific manner [43].

A large number of studies reported associations of either of the two proteins with diverse cellular compartments and different cellular structures such as microtubules, the centrosome, the endoplasmic reticulum, and the plasma membrane [44]. Most studies described cytoplasmic staining, especially intense in the perinuclear region and diminishing towards the plasma membrane [45,46,47,48]. The association of the NME/NDPKs with microtubules was proposed in 1984 by Nickerson and Wells [49]. Several groups confirmed a direct or indirect association of NME1 and/or NME2 with microtubules in human and other model systems [45,50,51,52,53], but other studies failed to draw any connection between NME proteins and the microtubuline network [47,54]. Further, Fan and coworkers reported NME1 to be a constituent of the SET-complex (endoplasmic reticulum (ER)-associated DNA repair complex) [33]. It was also suggested that NME2 facilitates COPII (Coat Protein Complex II) assembly essential for the ER-Golgi trafficking [55]. The results of our group using GFP-fused NME1 and NME2 proteins confirmed the co-localization of both proteins with the endoplasmic reticulum [46]. The association of the NDPK with the translation apparatus was proposed by Sonnnemann and Mutzel [56]. A number of authors demonstrated the association of NME proteins with intermediate filaments. For example, it was reported that the NDPK co-purifies with vimentin from different tissues [57]. Roymans and coworkers reported co-immunopreciptation of the rat ortholog of NME2 with vimentin in non-differentiated cells, whereas, both NME1 and NME2 were found to be associated with GFAP (glial fibrillary acidic protein), another intermediate filament protein [48]. Our group reported partial co-localization of either of the two NME proteins with vimentin, similar to Pinon and coworkers [45,46]. NME1 and NME2 have also been shown to localize at the plasma membrane. It was reported that ARF6-GTP (ADP-Ribosylation Factor 6-GTP) interacts with NME1 and recruits it to the membrane where it promotes dynamin activation [23]. NME2 was also found in this complex. The relatively recent studies of Romani et al. [58] and Boissan et al. [24] confirmed the involvement of NME/NDPKs with dynamin where the NDPK fuels dynamin with GTP and allows it to perform work with high thermodynamic efficiency, therefore, placing a portion of the proteins within the membrane.

Several authors reported the presence of either of the two subunits in the nucleus during different physiological processes [45,47,59]. Subramanian et al. and Murakami et al. [20,39] provided evidence that NME1 directly interacts with Epstein–Barr virus nuclear antigens and this interaction directs NME1 into the nucleus. Rayner and coworkers suggested that NME2 associates with ERβ (Estrogen Receptor β) protein when estrogen stimulation directs NME2 into the nucleus of smooth muscle cells [60]. Especially interesting are studies suggesting the participation of NME proteins in DNA damage repair. The first evidence to support the involvement of NME1 in base excision repair (BER) was observed in *Saccharomyces cerevisiae*, in which the yeast homolog of NME1, (YNK1), was shown to contribute to error-prone repair of DNA induced by UV light and etoposide [61]. In addition, NME1 was found to be overexpressed in a radiation dose-dependent manner together with APR1 (apurinic/apyrimidinic endonuclease-1), one of the crucial enzymes involved in BER. Using His pull-down assay and co-immunoprecipitation, it was demonstrated that APE1 interacts with NME1, suggesting the involvement of NME1 in BER in human pulmonary cancer cell line A549 [62]. Further, Jarret and coworkers [63] reported the role of NME1 in nucleotide excision repair (NER). It has also been demonstrated that NME1 participates in non-homologous end joining (NHEJ) of X-ray induced double-stranded breaks (DSB) by affecting the cell-cycle checkpoint signaling, DNA repair, and the MRN (a complex of three proteins, MRE11, RAD50 and NBS1 in humans, which functions as a major recognition and stabilization complex within the DSB repair) [64]. The very recent work of Xue and coworkers confirmed that NME1 primarily promotes NHEJ repair. They demonstrated that the knock-down of NME1 significantly compromises the repair of irradiation induced double-stranded breaks. NME1 localizes to sites of DNA damage while its downregulation impairs the recruitment of ligase IV (LIG4) responsible for end DSB ligation [65]. Although numerous studies reported the presence of both proteins mostly in the cytoplasm and occasionally in other compartments, the precise timing and mechanism by which they change their localization is still elusive. Additionally, it is not yet understood whether all potential NME functions require the hexameric form, and whether the isoenzyme composition varies in different cellular events and compartments.

The goal of our study was to examine a possible change in the localization of NME1 and NME2 proteins during 48 h after inducing DNA damage by live-cell imaging. Using FRET/FLIM, a well-established and sensitive technique for studying molecular interactions, we additionally examined the composition of NME1/NME2 oligomers in the nucleus and the cytoplasm in non-irradiated and gamma-irradiated cells. All experiments were performed using exogenous, fluorescently labeled NME proteins on irradiated and non-irradiated cells to determine the possible influence of gamma irradiation on the association of NME proteins into homomers and heteromers, and their localization. Our results reveal that NME1 and NME2 co-localize under all experimental conditions, both in non-irradiated and irradiated cells, in the cytoplasm and in the nucleus. After gamma irradiation, a fraction of both proteins simultaneously translocated into the cell nucleus. FRET experiments revealed a possible small shift of balance between the homomeric and heteromeric NME fractions after inducing DNA damage.

## 2. Results and Discussion

### 2.1. NME1 and NME2 Translocate to the Nucleus after Gamma Irradiation Treatment

Our previous study of the subcellular localization of NME1 and NME2 proteins, using fluorescent gene reporter system in order to specify their function and specific role in the cell, revealed that the two proteins co-localize regardless of the cell type [46]. The proteins were principally localized in the cytoplasm, but were also occasionally detected in the nucleus, especially in the late G1 phase. In this study, we used time-lapse microscopy to assess the in vivo distribution of both NME1 and NME2 proteins simultaneously, in non-irradiated and irradiated cells, in a 48-h period. For this purpose, we co-transfected HeLa cells with plasmids carrying EGFP-NME1 and mCherry-NME2. The non-irradiated cells were observed 24 h post-transfection during 48 h, while irradiated cells were observed one hour post-irradiation and 25 h post-transfection during 48 h. Due to cell manipulation after exposing the cells to gamma irradiation, it was impossible to observe irradiated cells immediately after induction of DNA damage using live-cell imaging. Therefore, the first hour post-irradiation was analyzed at fixed time points and on fixed cells transfected with plasmids carrying EGFP-NME1 and mCherry-NME2 using standard confocal microscopy. DNA damage was induced by subjecting HeLa cells to gamma irradiation (Co-60) 24 h after transfection with the selected plasmids producing fluorescently labeled proteins. The DNA damage was verified using Western blot analysis with the γH2AX antibody, which recognizes phosphorylation of histone protein γH2AX on Ser139. H2AX is phosphorylated at sites of double-stranded DNA breaks. Therefore, the upregulation of γH2AX indicated that the DNA damage was successfully induced in HeLa cells (Supplementary Figure S1). 

**Figure 1:**
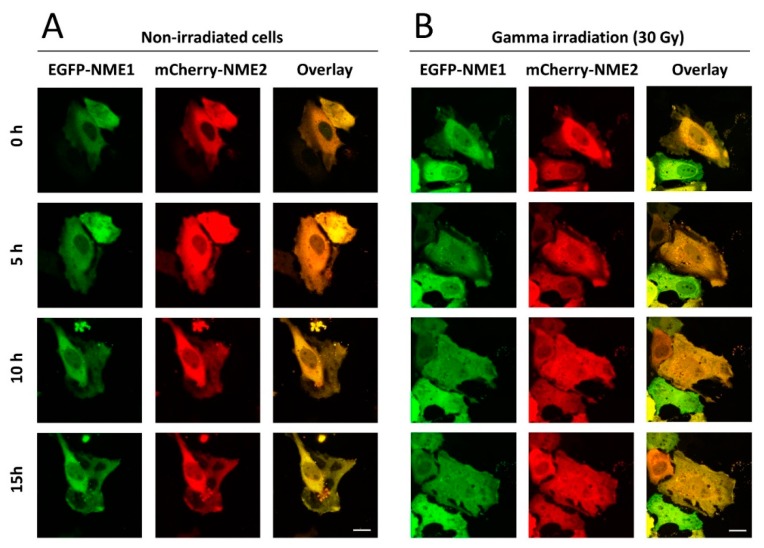
Time-lapse live-cell imaging showing subcellular localization of EGFP-NME1 and mCherry-NME2 in HeLa cells. The cells were observed under confocal microscope one day after transfection. (**A**) Non-irradiated cells. (**B**) Cells that were exposed to gamma irradiation (Co-60) of 30 Gy approximately one hour before the time point 0 h. Scale bar, 20 µm.

**Figure 2:**
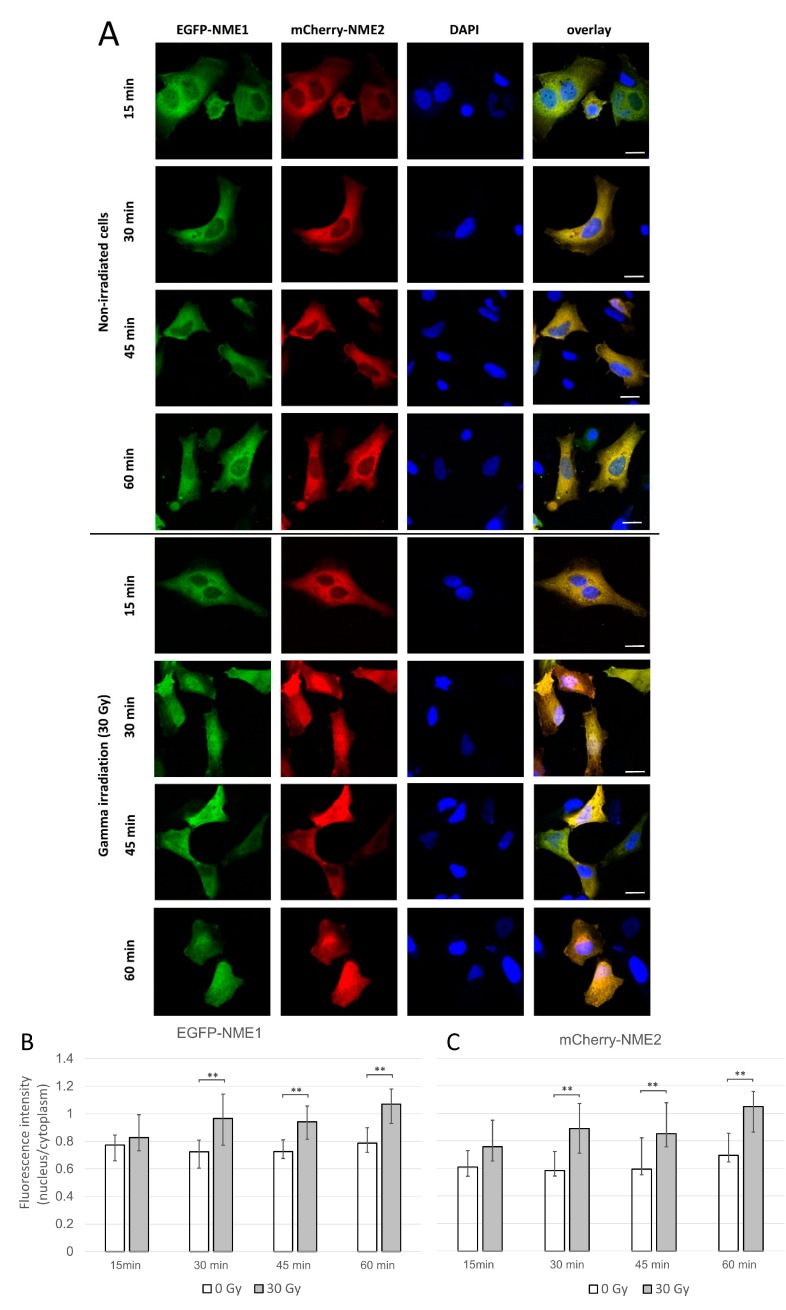
Localization of EGFP-NME1 and mCherry-NME2 in HeLa cells 24 h post-transfection in non-irradiated cells and cells exposed to gamma irradiation (Co-60) of 30 Gy. (**A**) Then, 24 h after transfection, cells were either irradiated or not and then fixed, after an additional period of 15, 30, 45, or 60 min. A representative image of non-irradiated and irradiated cells is shown for each time point. Scale bars, 20 µm. (**B**,**C**) Ratio of the fluorescence intensities of EGFP-NME1 (**B**) and mCherry-NME2 (**C**) in the cell nucleus and the cell cytoplasm is shown as median values and interquartile ranges. The difference between relative fluorescence intensities in the nuclei of irradiated and non-irradiated cells is statistically significant for periods of 30, 45, and 60 min. **: *p* ≤ 0.01.

Figure 1 displays typical localization of the fluorescently labeled NME proteins during 15 h in non-irradiated and irradiated cells using live-cell imaging. The results show that NME1 and NME2 co-localized and were present mostly in the cytoplasm of non-irradiated cells (Figure 1A). In irradiated cells, already one hour after irradiation when the imaging commenced, a massive translocation of both isoforms into the nuclei of the majority of cells could be seen (Figure 1B). Even 15 h post-irradiation, the fluorescence was comparable in its intensity to the corresponding cytoplasmic compartments. The enhanced nuclear staining of irradiated cells stayed visible in some cells even up to 24 h post-irradiation (data not shown). After that period, the survival of irradiated and even non-irradiated cells decreased. Importantly, EGFP-NME1 and mCherry-NME2 translocated to the nuclei simultaneously, which might indicate that the assembled oligomers entered the nucleus intact. This is consistent with the recent finding of a long-term stability of the purified NME1/NME2 hexamers [66]. Next, we performed experiments on fixed cells at fixed time points (15, 30, 45 and 60 min post-irradiation), which showed that the fluorescent NME proteins entered the nucleus already 30 min post-irradiation. The quantification of relative fluorescence intensity revealed that the staining was significantly stronger in the nuclei of irradiated cells compared to control, non-irradiated cells. This result substantiated our observation in live cells that both NME proteins entered the nucleus after irradiation (Figure 2A–C). 

The study of Jarett et al. [49] provided evidence that NME1 enters the nucleus of UV irradiated 793H1-FL8 cells several minutes post-induction while the nuclear signal reaches its maximum after one hour where it remains for about 12 h. Although this finding is, in principal, consistent with ours, UVR induces 6-4 photoproduct and other DNA-polymerase blocking lesions rather than double-stranded breaks, and, therefore, activates a different repair mechanism (NER). Sheng and coworkers suggested that NME1 enters the nucleus after DNA damage and possibly takes part in several DNA-repair mechanisms [64]. It has even been suggested by Park and coworkers that the nuclear localization of NME1 in head and neck squamous cell carcinoma could be associated with radiation resistance [67] and, therefore, serve as a strong radiation resistance predictor. Similar research has been reported on human nasopharyngeal carcinoma [68] and oral squamous cell carcinoma [69]. The results of our live-cell imaging experiments clearly showed that the two NME subunits are present mostly in the cytoplasm of non-irradiated cells. After irradiation, both proteins enter the nucleus simultaneously. While the presence of NME1 and NME2 in the nucleus has been thoroughly documented [42,47], their simultaneous, although, possibly independent, entrance after DNA damage induction is quite puzzling. Although the involvement of NME1 in several DNA repair mechanisms is fairly well established, there has been very little evidence that NME2 participates in any of the DNA repair processes [70]. Taken together, it remains unclear whether NME2 takes an active part in the repair of irradiation-induced DNA damage. 

Due to the cytotoxic effects of transfection and irradiation treatments, we were unable to track the potential return of the NME proteins into the cytoplasm in irradiated cells, but the return time seems to be even 24 h in some cells, under our conditions (data not shown). It is well known that the nuclear double membrane contains pore complexes which allow the transfer of ions, small molecules, and small macromolecules in and out of the nucleus by simple diffusion. Most proteins, however, use active transport to enter or exit the nucleus, a process that requires ATP and a nuclear localization signal (NLS) or a nuclear export signal (NES) in their amino acid sequence. The NME proteins lack NLS, which implies that they are transported into the nucleus by a carrier protein [71]. A demand for an increased supply of the NME subunits into the nuclei of irradiated cell might appear plausible, since the enzyme is required for synthesizing NTPs and dNTPs for DNA repair or possible transcription of the necessary genes after DNA damage. This would justify the massive entrance of both proteins into the nucleus upon irradiation that we have observed in our experiments. However, nucleotides can easily enter the nucleus through the nuclear pore complexes, which makes the potential, energetically demanding, transport of the NME proteins solely for that purpose less probable. It is possible that the amplified NME1/2 signal in the nucleus of irradiated cells indicates another individual function of the NME1 and/or NME2 induced by DNA damage. Local fueling of certain nuclear proteins with a specific type of nucleotides is a valid possibility, since similar actions of the NME family members have been described [24,72]. However, the massive, simultaneous movement of both NME proteins into the nucleus favors the hypothesis that they are both engaged in the same function connected to DNA damage repair.

### 2.2. Choosing the Optimal FRET Reporter Orientation for Estimating Fractional FRET Population of Homomers and Heteromers

Förster resonance energy transfer (FRET) is a phenomenon that describes energy transfer through nonradiative dipole–dipole coupling between a donor and an acceptor molecule in close proximity (<10 nm) [73]. When a fluorescent donor experiences FRET, its bulk fluorescence intensity and the lifetime of its excited state decrease. FRET can be used to monitor protein–protein interactions (PPIs) in biological systems such as living cells, tissues, and organisms [74,75,76]. A widely used method to measure FRET is fluorescence lifetime imaging microscopy (FLIM). In FRET-FLIM we measure the donor’s excited state decay in the presence of an acceptor [77], i.e., estimate the change in the donor’s lifetime induced by the acceptor. The main advantage of the FRET-FLIM method, compared to traditional biochemical techniques for monitoring of PPIs, such as co-immunoprecipitation (co-IP), is that it supports in vivo studies, is highly sensitive and specific, and can provide information on the location of the interaction [78]. In this study, we used EGFP (the donor molecule) and mCherry (the acceptor molecule) for labeling NME proteins. EGFP and mCherry represent a suitable FRET pair due to a relatively long fluorescence lifetime of EGFP (2.4 ns), and a relatively large overlap between donor’s emission and acceptor’s excitation spectra [79]. The FRET-FLIM method that we used in this article estimated the fraction of fluorescent donor molecules in the sample that experience FRET. This fraction of donor molecules that experienced FRET can be simplistically interpreted as a measure of the fraction of EGFP-labeled NME proteins that reside in the same oligomer as mCherry-labeled NME proteins. We designated this “fractional FRET population” by *D_F_*.

For FRET-FLIM measurements, mCherry was fused N-terminally with both NME1 and NME2 (mCherry-NME1 and mCherry-NME2), whereas EGFP was fused with both proteins either N-terminally (EGFP-NME1 and EGFP-NME2) or C-terminally (NME1-EGFP and NME2-EGFP). It follows that we can use two combinations of fusion proteins for estimating *D_F_* for either of the two homomers, NME1/NME1 and NME2/NME2, and four combinations of fusion proteins for estimating *D_F_* for the heteromer NME1/NME2. As the first step, we decided to determine which combination of fusion proteins represents the best reporter for each oligomer, resulting in the largest *D_F_*. Namely, the relative position of fluorophores with respect to the labeled proteins, being fused either to the N-terminus or the C-terminus, can contribute differently to the overall FRET effect. The largest *D_F_* provides the largest dynamic range for the detection of possible changes of *D_F_* induced by experimental manipulation.

**Figure 3:**
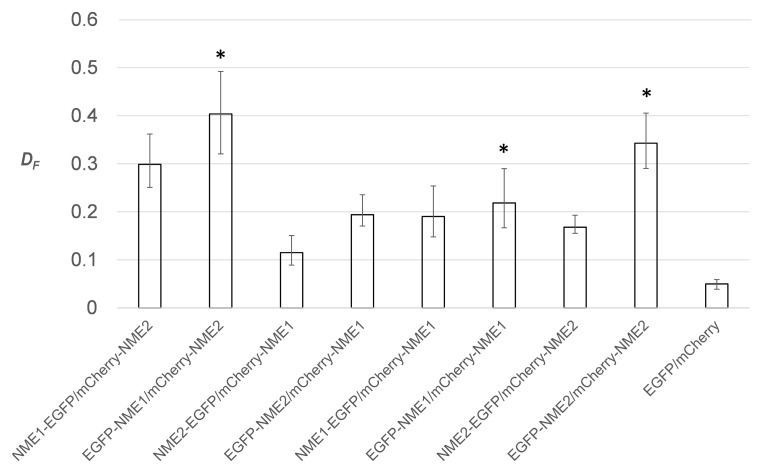
Fractional FRET population (*D_F_*) for different combinations of fluorescently labeled NME proteins: NME1/NME2, NME1/NME1, and NME2/NME2 (see main text for explanation). Donor molecule (EGFP) was fused either to the N-terminus or the C-terminus of an NME protein, while the acceptor molecule (mCherry) was always fused to the N-terminus of an NME protein. *D_F_* is shown as the median and the interquartile range. The star sign (*) indicates the highest *D_F_* for each of the three combinations of NME proteins.

*D_F_* was estimated for each of the 8 possible donor–acceptor combinations of fusion proteins based on the FRET-FLIM measurements conducted using 18 cells from each group. The obtained results showed that in all cases NME proteins with EGFP attached to their N-terminus exhibited the highest fractional FRET population *D_F_* (Figure 3). The residual background *D_F_* was estimated using the control sample, i.e., the cells expressing EGFP and mCherry alone, and amounted to approximately 0.05. Although the C and the N termini in both NME proteins are positioned on the surface of the molecule in close vicinity to each other, it was published that the C-terminus of a NME peptide stabilizes the NDPK hexameric structure [80]. Fusing the fluorophore to the C-terminus of either of the two proteins could, therefore, interfere with the stabilization of the hexameric structure and diminish the overall *D_F_*, as observed (Figure 3). All the following experiments were, therefore, conducted with the fluorophores fused to the N termini of NME proteins. 

Since the concentration of the exogenous proteins produced under a potent CMV promoter exceeded by far the level of the endogenous proteins in a transfected cell [81,82,83,84,85,86,87], the participation of endogenous proteins in the oligomer formation was considered negligible. Also, it is generally accepted that NME proteins are present in the cell as hexamers. However, since we cannot exclude the presence of other oligomeric forms under our experimental conditions, in this manuscript we are using the terms oligomer, homomer, and heteromer rather than hexamer, homohexamer, and heterohexamer.

### 2.3. FRET/FLIM Experiments Indicate that DNA Damage Slightly Changes the Balance between the Oligomers in Cellular Compartments

**Figure 4:**
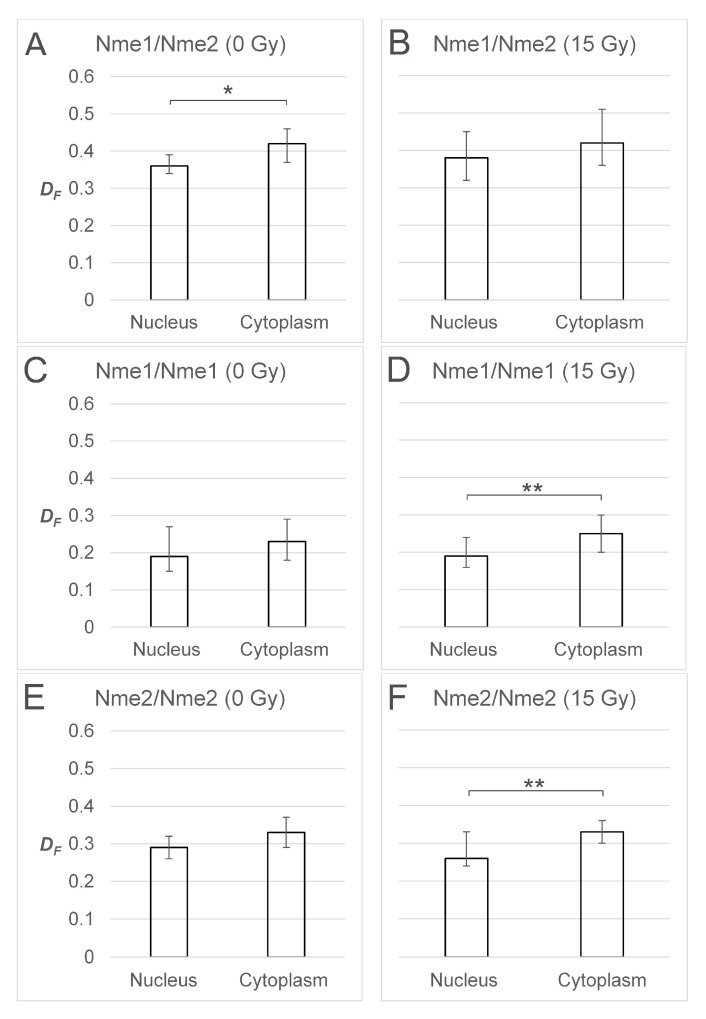
Comparison of *D_F_* between the nucleus and the cytoplasm in non-irradiated and irradiated cells expressing three pairs of fluorescently labeled NME proteins. Transfected cells were not irradiated (0 Gy) or irradiated with gamma radiation (15 Gy). (**A**,**B**) Comparison of *D_F_* between the nucleus and the cytoplasm in non-irradiated (**A**) and irradiated (**B**) cells expressing EGFP-NME1 and mCherry-NME2. (**C**,**D**) Comparison of *D_F_* between the nucleus and the cytoplasm in non-irradiated (**C**) and irradiated (**D**) cells expressing EGFP-NME1 and mCherry-NME1. (**E**,**F**) Comparison of *D_F_* between the nucleus and the cytoplasm in non-irradiated (**E**) and irradiated (**F**) cells expressing EGFP-NME2 and mCherry-NME2. *D_F_* is shown as the median and the interquartile range. Statistical analysis shows that differences of *D_F_* between the nucleus and the cytoplasm (**A**,**D**,**F**) are statistically significant. *: *p* ≤ 0.05, **: *p* ≤ 0.01.

To assess the possible difference in the compartmentalization of different protein assemblies, we compared the fractional FRET population, *D_F_*, between the nucleus and the cytoplasm in non-irradiated and irradiated cells expressing one of the three pairs of fluorescently labeled proteins (NME1/NME2, NME1/NME1, NME2/NME2). It was apparent that the *D_F_* was slightly larger in the cytoplasm than in the nucleus in all of the NME protein combinations and under both conditions, which indicates a systemic effect, probably on the FRET process itself. The cytoplasmic *D_F_* in the case of the NME1/NME2 was statistically significantly larger than the corresponding nuclear *D_F_* in non-irradiated cells (Figure 4A). The statistical significance of this difference was lost after irradiation, but we noted an increased variation of *D_F_* in irradiated cells (Figure 4B). On the contrary, the difference between the cytoplasmic and the nuclear *D_F_* was not statistically significant in non-irradiated cells expressing either of the labeled homomers, NME1/NME1 and NME2/NME2, but it became statistically significant after irradiation (Figure 4C–F). 

On the basis of the statistical significance alone, one could argue that in the irradiated cells the balance during the NME oligomer assembly or relocation shifts slightly in favor of homomers in the cytoplasm and heteromers in the nucleus. This phenomenon can be a result of a selective translocation of specific NME oligomers after irradiation, but it can also indicate enhanced re-assembly of NME heteromers in the nucleus. However, no statistically significant difference was determined when *D_F_* values were compared between non-irradiated and irradiated cellular compartments for any particular oligomer (Supplementary Figure S2). As already mentioned, NME proteins in eukaryotes exert their NDPK function only in the form of hexamers. Gilles and coworkers obtained results that favor the hypothesis that NDP kinases form isoenzymes by random association of the two subunits, NME1 and NME2 [4]. Using native mass spectrometry, Potel et al. showed that purified endogenous NME1 and NME2 oligomerize following the statistical rule and, therefore, form primarily heterohexamers [66]. The distribution and abundance of different hexamers follows the hypergeometrical distribution, indicating that the probability of incorporation into an oligomer is equal for NME1 and NME2. In the case of the equal number of both protein species, both possible homohexameric assemblies, NME1_6_ and NME2_6_, are present in marginal proportions, representing less than 2% of the total hexameric population. A higher abundance of either of the homohexamers can thus occur solely as a consequence of a higher stoichiometry of one or the other isoform and is directly dependent on their relative transcription levels. The observed slight changes in the relative *D_F_* between the nucleus and the cytoplasm upon gamma irradiation might indicate a shift in the relative abundance of the two isoforms or a change in their interaction kinetics. Either effect would increase the fraction of homomers in one of the cell compartments.

**Figure 5:**
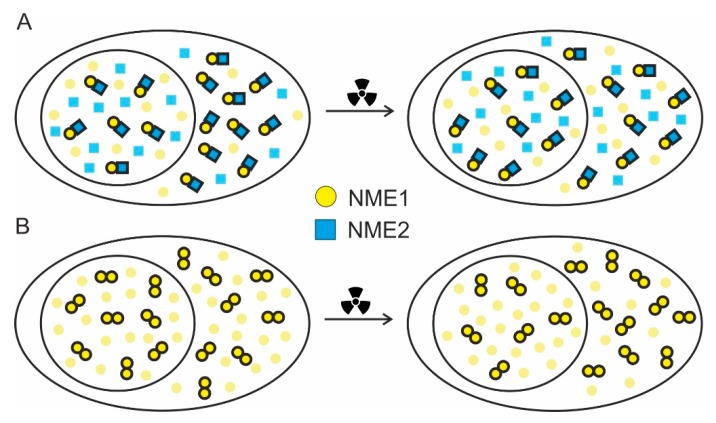
Schematic interpretation of the changes in *D_F_* determined by FRET/FLIM experiments and shown in Figure 4. (**A**) Before irradiation, *D_F_* for fluorescently labelled NME1 and NME2 is significantly larger in the cytoplasm than in the nucleus, indicating more NME1/NME2 heteromers in the cytoplasm than in the nucleus. After irradiation, this difference is rendered insignificant. This panel corresponds to Figure 4A,B. (**B**) Before irradiation, *D_F_* for fluorescently labelled NME1 is not distinguishable between the cytoplasm and the nucleus. After irradiation, *D_F_* becomes significantly larger in the cytoplasm than in the nucleus, indicating more NME1 homomers in the cytoplasm than in the nucleus. This panel corresponds to Figure 4C,D. An equivalent interpretation applies to Figure 4E,F, corresponding to oligomerization of NME2. Oligomerization state of NME proteins is simplistically shown as being either dimeric or monomeric, whereas in reality different hexameric configurations probably dominate. In both panels, the differences between the cytoplasm and the nucleus are exaggerated for clarity.

It could be speculated that the balance of the homomeric versus heteromeric populations between cellular compartments is more important than their absolute levels. The difference in the balance could support the theory that in certain cases, in this case DNA damage, a fraction of NME1 and NME2 execute some discrete, diverse functions in the homomeric state. Since this shift in balance was not large, we presumed that the fraction of the NME1 or NME2 proteins engaged in any other function, other than the NDPK nucleotide supply function, after DNA damage is rather small. A simplified schematic representation of the results obtained by FRET/FLIM experiments can be seen in Figure 5.

In order to independently corroborate protein–protein interactions obtained by FRET/FLIM experiments, we performed co-immunoprecipitation of NME1 and NME2 proteins from the nuclear and the cytosolic fraction after cell fractionation. For that purpose, we co-transfected the cells with NME1-FLAG (FLAG - short octapeptide tag) and NME2 protein and performed pull-down experiments with anti-FLAG M2 agarose separately on nuclear and cytosolic fractions. Our results showed high expression levels of both proteins in the cytosolic fraction and lower expression levels of both proteins in the nuclear fraction (Supplementary Figure S3). We were able to detect the interaction of exogenous NME1-FLAG and NME2 proteins in both fractions, even though it was substantially weaker in the nuclear fraction. Our results indicate that the use of FRET/FLIM method for comparing the extent of protein–protein interactions in different cellular compartments is more robust, with respect to the variations of protein expression levels between the compartments, than co-immunoprecipitation. 

Lastly, this is the first study that uses the FRET/FLIM method to assess the oligomerization properties of the two NDPK/NME protein subunits. We showed that fluorophores fused to the N-termini of both NME polypeptides produce the largest FRET effect upon their interaction and, therefore, should be used for similar studies. 

## 3. Materials and Methods

### 3.1. Cell Lines

HeLa (ATCC^®^ CCL-2™) cells were grown in DMEM (Dulbecco’s Modified Eagle Medium) (Gibco, Thermo Fisher Scientific, Waltham, MA, USA) supplemented with 10% fetal bovine serum (Thermo Fisher Scientific, Waltham, MA, USA), 1% streptomycin-penicillin (Sigma Aldrich, St. Louis, MO, USA), 1 mM sodium pyruvate (Life Technologies, Carlsbad, CA, USA) and 2 mM L-glutamine (Sigma Aldrich, St. Louis, MO, USA). Cells were maintained in a humidified chamber at 37 °C under 5% CO_2_ atmosphere. HeLa cell line was tested mycoplasma-free.

### 3.2. Plasmids

The pEGFPN1-NME1 and pEGFPN1-NME2 were obtained courtesy of Dr. Marie-Lise Lacombe (French Institute of Health and Medical Research, Centre de Recherche St Antoine, U938, Paris, France). The pcDNA3-NME1-FLAG was obtained as described before [88]. The pEGFPC1-NME1 and pEGFPC1-NME2 vectors were constructed by subcloning from pcDNA3 vectors containing cDNAs from either of the two genes (obtained courtesy of Dr. Marie-Lise Lacombe, French Institute of Health and Medical Research, Centre de Recherche St Antoine, U938, Paris, France) into pEGFPC1 vector (Clontech, Kyoto, Japan). In brief, the pcDNA3-NME vectors were digested with EcoRI/XhoI and ligated with EcoRI and Sal I sites into pEGFPC1 expression vector. For cloning into pmCherry, the PCR-generated cDNA for NME1(5’GTCTAGCTCGAGTAATGGCCAACTGTGAGCG 3′ and 5′ CTAGACGAATTCTCATCATTCATAGATCC 3′) and NME2 (5’GTCTAGCTCGAGTAATGGCCAACCTGGAGC 3′ and 5′ CTAGACGAATTCTTATTATTCATATACCCAGTCATGAGC 3′) were digested with XhoI and EcoRI and ligated with XhoI/EcoRI sites of pmCherryC1 expression vector. The NME1 and NME2 fragments were amplified using EmeraldAmp MAX PCR Master Mix (Clontech, Kyoto, Japan). Plasmid constructs were verified by sequencing (ABI PRISM 3100-Avant Genetic Analyzer, Applied Biosystems, Foster City, CA, USA) using ABI PRISM BigDye Terminator v.3.1 Ready Reaction Cycle Sequencing Kit (Applied Biosystems, Foster City, CA, USA).

### 3.3. Transfection

For FRET-FLIM analysis and live-cell imaging, 10^5^ cells/well were seeded 24 h prior to transfection in a four-chamber glass-bottom dish (Cellvis, Mountain View, CA, USA). For fixed samples, 5.5 × 10^4^ cells were seeded in an 8-well chamber slide (Ibidi, Gräfelfing, Germany). For co-immunoprecipitation experiments, 2 × 10^6^ cells were seeded in 100-mm Petri dishes (BD Biosciences, Franklin Lakes, NJ, USA). About 90% confluent HeLa cells were transfected using Lipofectamine 2000 (Invitrogen, Thermo Fisher Scientific, Waltham, MA, USA) transfection reagent with 1.25 µg, 1.5 µg, and 12 µg of total plasmid DNA, respectively, according to manufacturer’s protocol. For co-transfection experiments, two plasmids in 1:1 ratio were used but with the equal total amount of DNA.

### 3.4. DNA Damage

DNA damage was induced by gamma ray emitter cobalt-60 (Co-60). Twenty-four hours post-transfection, the cells were irradiated either with 15 Gy (for FRET/FLIM analysis) by dose rate of 2 Gy/min or with 30 Gy (for confocal imaging and co-immunoprecipitation experiments) by dose rate of 4 Gy/min. The media was changed immediately after irradiation and the cells subjected to confocal imaging or protein isolation. For Western blot analysis after irradiation, the cells were harvested from a 100-mm Petri dishes (BD Biosciences, Franklin Lakes, NJ, USA). The non-irradiated and irradiated cells were collected by washing with PBS and scraping. Irradiated cells were collected 24 h after irradiation. Proteins were extracted in Dulbecco’s phosphate buffer saline (DPBS) (Sigma Aldrich, St. Louis, MO, USA) completed with protease inhibitors (complete, mini, EDTA (ethylenediaminetetraacetic acid)-free; Roche Diagnostics, Basel, Switzerland). Pellets were sonicated (1 mm probe, 2 × 10 sec). Protein concentration was determined by the Pierce BCA Protein Assay Kit (Pierce, Thermo Fisher Scientific, Waltham, MA, USA).

### 3.5. Co-immunoprecipitation

Co-immunoprecipitation experiments were done on the nuclear and the cytosolic fraction after transfection and induction of DNA damage. Nuclear and cytoplasmic proteins were extracted using Nuclear Extract Kit (Abcam, Cambridge, UK, ab221978) according to manufacturer’s protocol. Protein concentration was determined by Pierce BCA Protein Assay Kit (Pierce, Thermo Fisher Scientific, Waltham, MA, USA). Co-immunoprecipitation was performed using ANTI-FLAG^®^ M2 Agarose Affinity Gel (Sigma, Munich, Germany). Agarose beads were pre-equilibrated by washing with TENN buffer (50 mM Tris pH 7.4, 150 mM NaCl, 5 mM EDTA, 0.5% NP-40 (*v/v*)) three times. The amount of 100 µg of total proteins was transferred in tube with pre-equilibrated beads and it was incubated over night at 4 °C on a rocking platform. Total cell lysate of non-transfected cells was used as a control for immunoprecipitation (CTRL). The next day, beads were washed with PBS and PBS/0.05% Tween-20. Amount of 20 µg of total proteins was used as input for nuclear and cytosolic fraction (INP N and INP C). Proteins were denatured and loaded onto 12% SDS-polyacrylamide gel. 

### 3.6. Western Blot Analysis

Proteins were separated on 12% SDS-polyacrylamide gel (co-immunoprecipitation) and 15% SDS-polyacrylamide gel (DNA damage) and transferred to a nitrocellulose membrane (Merck Millipore, Burlington, MA, USA). The antibodies used in this study were as follows: Primary mouse anti-NME2 (Kamiya Biomedical, Tukwila, WA, USA, KM1121, 1:1000), primary mouse anti-FLAG (Sigma Aldrich, St. Louis, MO, USA, SLBN5629V, 1:500), primary rabbit anti-histone H3 (Abcam, Cambridge, UK, ab1791, 1:1000), primary mouse anti-GAPDH (from apoptosis WB antibody cocktail, Abcam, Cambridge, UK, ab110415, 1:500), primary rabbit anti-γH2AX (Abcam, Cambridge, UK, ab11174 1:2000), primary mouse anti-β-actin (Proteintech, Rosemont, IL, USA, 60008-1-1g, 1:3000), secondary HRP (horseradish peroxidase)-conjugated anti-rabbit (Cell signaling, Danvers, MA, USA, 1:3000) and secondary HRP-conjugated anti-mouse (GE Healthcare, London, UK, 1:3000). Proteins were visualized using Western Lightning Chemiluminescence Reagent Plus (Perkin Elmer, Waltham, MA, USA) on Alliance 4.7 imaging system (UVItec, Cambridge, UK). 

### 3.7. Confocal Imaging of Live and Fixed Cells

Confocal microscopy was performed using Leica TCS SP8 X FLIM microscope equipped with a HC PL APO CS2 63×/1.40 oil objective, 405-nm diode laser, and a supercontinuum excitation laser (Leica Microsystems, Wetzlar, Germany). Stage-top environmental control system was used for time-lapse live-cell imaging to maintain temperature at 37 °C and Leibovitz’s L-15 medium (Thermo Fisher Scientific, Waltham, MA, USA) was used to support cell growth in the environment without CO_2_ equilibration.

For analysis of the first hour post-irradiation, 24-h post-transfection cells were washed three times in phosphate-buffered saline (PBS), fixed in 2% paraformaldehyde (PFA) for 15 min and washed three times in PBS. After fixation, cells were mounted in mounting medium (DAKO, Glostrup, Denmark) supplemented with 1 µg/mL 4′,6-diamidino-2-phenylindole (DAPI) (Sigma, Munich, Germany) for nuclear staining. 

The excitation wavelengths and detection ranges used for confocal imaging of live and fixed cells were as follows: 488 nm and 500–550 nm for EGFP; 575 nm, 588–650 nm for mCherry; and 405 nm and 430–500 nm for DAPI. The hybrid (HyD) detectors were operated in the gated mode in order to suppress parasite reflection from the bottom glass surface of the cell-culture dish.

Fluorescence intensity was quantified using the function *Measure* in ImageJ (Rasband, W.S., U. S. National Institutes of Health, Bethesda, MD, USA, https://imagej.nih.gov/ij/). Measurements were performed on 20 non-irradiated and 20 irradiated cells for each time point (15, 30, 45 and 60 min).

### 3.8. FRET-FLIM Analysis

Fluorescence lifetime measurements were performed with Leica TCS SP8 X FLIM confocal microscope (Leica Microsystems, Wetzlar, Germany), using the time-correlated single photon counting (TCSPC) module (PicoQuant, Berlin, Germany). For photon detection, spectral FLIM photomultipliers (SP) were used. Data analysis was performed using SymPhoTime64 software (PicoQuant, Berlin, Germany). Photon detection for the entire single cell and the cell cytoplasm was set to 100 repetitions, and for the cell nuclei to 300 repetitions, with the peak count rate adjusted between 200 and 500 kcounts/s. The donor fluorophore, EGFP, was excited at 488 nm using a pulsed supercontinuum excitation laser with the pulse frequency set to 40 MHz. Temperature control and CO_2_ equilibration were performed as described above (see live-cell imaging). 

To obtain the fluorescence lifetime of the donor-only sample, cells were transiently transfected with plasmid carrying donor FRET molecule (EGFP protein fused with NME1 or NME2 protein). Donor fluorescence lifetime, $\tau_{0}$, was then estimated by fitting a mono-exponential model of fluorescence decay:

$$N\left(t\right)=Ae^{-t/\tau_{0}}$$

to experimental decay curves. This value of $\tau_{0}$ served as a fixed parameter in subsequent analysis of FRET samples. To obtain the fluorescence lifetime of donor in the presence of acceptor, cells were transiently transfected with various combinations of plasmids encoding either donor EGFP or acceptor mCherry fused with NME1 or NME2 proteins. For analyzing the donor fluorescence lifetime in FRET experiments, we used a double-exponential model of fluorescence decay:

$$N\left(t\right)=A_{1}e^{-t/\tau_{0}}+A_{2}e^{-t/\tau_{q}},$$

where amplitudes $A_{1}$ and $A_{2}$ and quenched lifetime $\tau_{q}$ were free parameters of the fit. The fraction of donor molecules that experience FRET, $D_{F}$, was calculated as:

$$D_{F}=A_{2}/\left(A_{1}+A_{2}\right).$$

For each pair of fusion proteins, measurements were performed on 20 non-irradiated cells and 24 irradiated cells, $D_{F}$ was determined for each cell, and then averaged (median ± interquartile range). Statistical comparison between samples was performed using the Kruskal–Wallis test implemented in MATLAB software (MathWorks, Natick, MA, USA,) (function *kruskalwallis*). The post-hoc Tukey–Kramer test was applied for multiple comparisons (function *multcompare*). The significance level was set to 5%.

## Supplementary Materials

Supplementary materials can be found at https://www.mdpi.com/1422-0067/21/7/2363/s1.

## Abbreviations

BER	Base Excision Repair
APE1	Apurinic/Apyrimidinic Endonuclease 1
NHEJ	Non-Homologous End Joining
CMV	Cytomegalovirus
DSBR	Double-Stranded Break Repair
EBNA 1–3C	Epstein–Barr Nuclear Antigen 1–3C
EGFP	Enhanced Green Fluorescent Protein
ERβ	Estrogen Receptor β
FLIM	Fluorescence-Lifetime Imaging Microscopy
FRET	Fluorescence Resonance Energy Transfer
ICAP1α	Integrin Cytoplasmic Domain-Associated Protein 1α
ER	Endoplasmic Reticulum
COP II	Coat Protein Complex II
GFAP	Glial Fibrillary Acidic Protein
ARF6	ADP-Ribosylation Factor 6
KSR	Kinase Suppressor of Ras
MDM2	Mouse Double Minute 2 Homolog
MIF	Macrophage Migration Inhibitory Factor
NDP	Nucleoside Diphosphate
NDPK	Nucleoside Diphosphate Kinase
NER	Nucleotide Excision Repair
NES	Nuclear Export Signal
NLS	Nuclear Localization Signal
NTP	Nucleoside Triphosphate
PDGF	Platelet-Derived Growth Factor
STRAP	Serine/Threonine Kinase Receptor Associated Protein
TRF1	Telomeric Repeat Binding Factor 1
UVR	Ultraviolet Radiation
YNK1	Yeast Homolog of NME1
