# Supplementary material for: The Subcellular Localization and Oligomerization Preferences of NME1/NME2 upon Radiation-Induced DNA Damage
Source: Int J Mol Sci. 2020 Mar 29;21(7):2363. doi: 10.3390/ijms21072363 (PMC7177722; doi:10.3390/ijms21072363)
Supplement: Supplementary file 1 [file ijms-21-02363-s001.pdf]

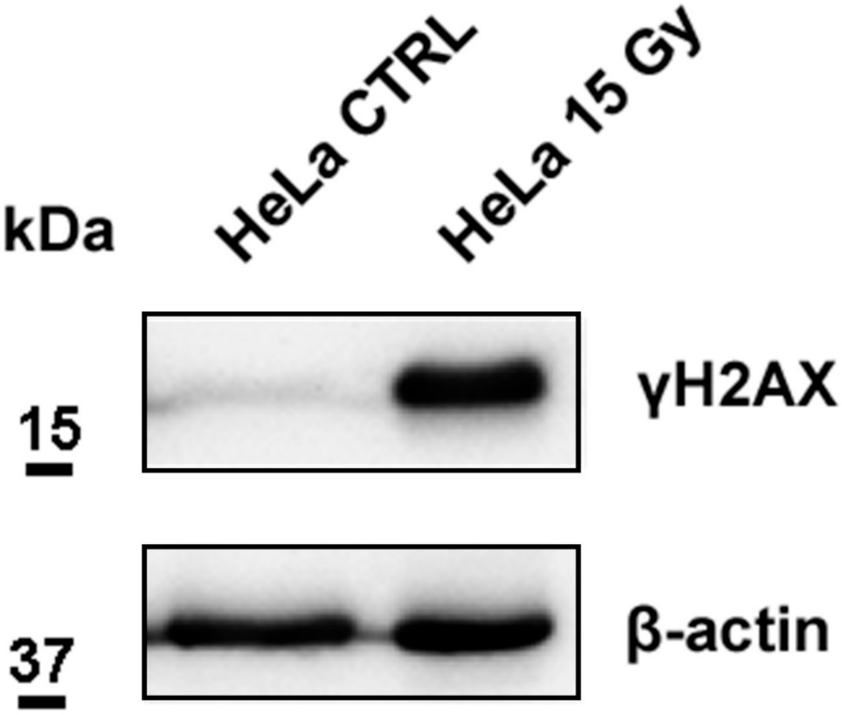

Supplementary Figure S1. Western blot analysis performed on HeLa cells lysates, before and after exposure to gamma irradiation of 15 Gy. Antibody against γH2AX specifically recognizes phosphorylation of histone protein γH2AX on Ser139. The elevated γH2AX shows the emergence of double-stranded DNA breaks.

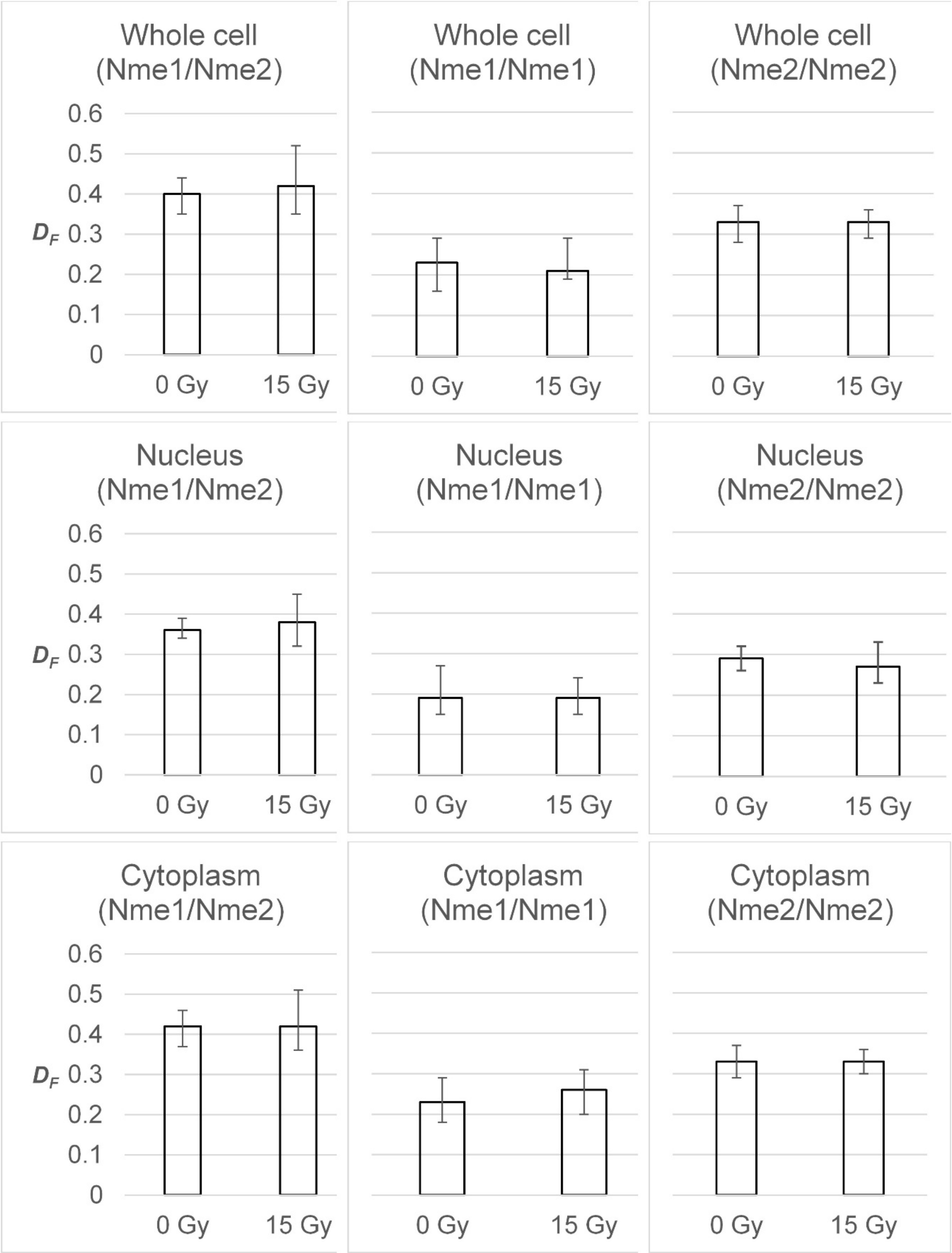

Supplementary Figure S2. The influence of gamma irradiation on D_F in each cellular compartment (nucleus and the cytoplasm), of cells expressing three pairs of fluorescently labeled NME proteins. Non-irradiated transfected HeLa cells (0 Gy) and gamma irradiated HeLa cells (15 Gy) are compared. D_F is shown as the median and the interquartile range. None of the pairwise differences are statistically significant.

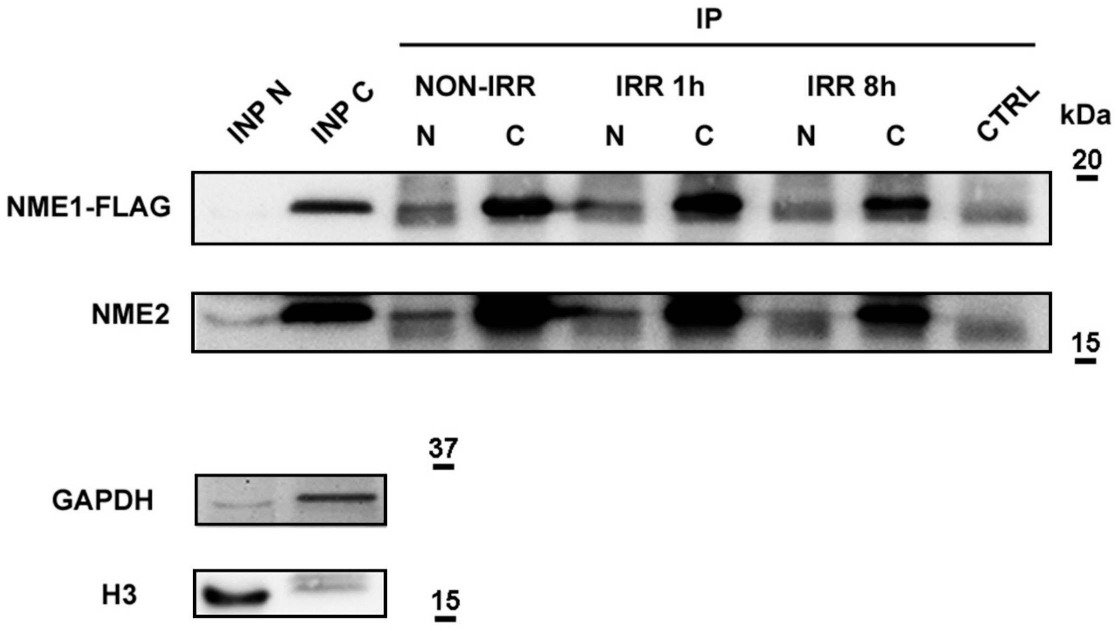

Supplementary Figure S3. Coimmunoprecipitation of NME1-FLAG and NME2 proteins in the nucleus and the cytoplasm. Coimmunoprecipitation was detected in non-irradiated cells (NON-IRR) and cells subjected to gamma irradiation (30 Gy) collected 1 and 8 h after irradiation (IRR 1h and IRR 8h, respectively). Anti-GAPDH antibody was used as a marker for cytosolic fraction and anti-histone H3 as a marker for nuclear fraction. NME1-FLAG protein was visualized with anti-FLAG antibody and NME2 protein with anti-NME2 antibody.
